# Comparative genomics and evolution of regulons of the LacI-family transcription factors

**DOI:** 10.3389/fmicb.2014.00294

**Published:** 2014-06-11

**Authors:** Dmitry A. Ravcheev, Matvei S. Khoroshkin, Olga N. Laikova, Olga V. Tsoy, Natalia V. Sernova, Svetlana A. Petrova, Aleksandra B. Rakhmaninova, Pavel S. Novichkov, Mikhail S. Gelfand, Dmitry A. Rodionov

**Affiliations:** ^1^Research Scientific Center for Bioinformatics, A.A. Kharkevich Institute for Information Transmission Problems, Russian Academy of SciencesMoscow, Russia; ^2^Faculty of Bioengineering and Bioinformatics, Moscow State UniversityMoscow, Russia; ^3^Lawrence Berkeley National Laboratory, Genomics DivisionBerkeley, CA, USA; ^4^Department of Bioinformatics, Sanford-Burnham Medical Research InstituteLa Jolla, CA, USA

**Keywords:** bacteria, transcription factors, regulons, sugar metabolism, comparative genomics

## Abstract

DNA-binding transcription factors (TFs) are essential components of transcriptional regulatory networks in bacteria. LacI-family TFs (LacI-TFs) are broadly distributed among certain lineages of bacteria. The majority of characterized LacI-TFs sense sugar effectors and regulate carbohydrate utilization genes. The comparative genomics approaches enable *in silico* identification of TF-binding sites and regulon reconstruction. To study the function and evolution of LacI-TFs, we performed genomics-based reconstruction and comparative analysis of their regulons. For over 1300 LacI-TFs from over 270 bacterial genomes, we predicted their cognate DNA-binding motifs and identified target genes. Using the genome context and metabolic subsystem analyses of reconstructed regulons, we tentatively assigned functional roles and predicted candidate effectors for 78 and 67% of the analyzed LacI-TFs, respectively. Nearly 90% of the studied LacI-TFs are local regulators of sugar utilization pathways, whereas the remaining 125 global regulators control large and diverse sets of metabolic genes. The global LacI-TFs include the previously known regulators CcpA in Firmicutes, FruR in Enterobacteria, and PurR in Gammaproteobacteria, as well as the three novel regulators—GluR, GapR, and PckR—that are predicted to control the central carbohydrate metabolism in three lineages of Alphaproteobacteria. Phylogenetic analysis of regulators combined with the reconstructed regulons provides a model of evolutionary diversification of the LacI protein family. The obtained genomic collection of *in silico* reconstructed LacI-TF regulons in bacteria is available in the RegPrecise database (http://regprecise.lbl.gov). It provides a framework for future structural and functional classification of the LacI protein family and identification of molecular determinants of the DNA and ligand specificity. The inferred regulons can be also used for functional gene annotation and reconstruction of sugar catabolic networks in diverse bacterial lineages.

## Introduction

Evolution of regulatory interactions in bacteria can be approached from three directions. The first approach is the comparative analysis of regulation of a functional system, e.g., a metabolic pathway, in a variety of species. Such analysis demonstrates high flexibility of regulatory interactions even in closely related species, with expansion, contraction, and merging of regulons or a complete change of regulators (Manson McGuire and Church, 2000; McCue et al., 2001; Tan et al., 2001; Gelfand, 2006; Rodionov et al., 2006; Ravcheev et al., 2007; Kazakov et al., 2009; Suvorova et al., 2011, 2012). The second approach is to consider a taxon of a relatively low level (genus or family) and to use comparative genomics to predict as many regulatory interactions as possible. This has been done for γ-Proteobacteria from the *Shewanella* genus (Rodionov et al., 2011); Firmicutes closely related to *Bacillus subtilis* (Leyn et al., 2013) and *Staphylococcus aureus* (Ravcheev et al., 2011); two families of lactic acid bacteria from the Lactobacillales order (Ravcheev et al., 2013b); hyperthermophilic bacteria related to *Thermotoga maritima* (Rodionov et al., 2013); human gut habitant *Bacteroides thetaiotaomicron*; and related organisms (Ravcheev et al., 2013a). An important side product of such studies is functional annotation of hypothetical proteins by assigning them, via co-regulation, to known metabolic pathways and other functional subsystems (Rodionov, 2007; Gelfand and Rodionov, 2008).

The third approach, implemented here, is to consider a family of transcription factors (TFs) and then identify binding motifs for as many TFs as possible. This is mainly motivated by the desire to analyze the structure of protein–DNA interactions and the co-evolution of TFs and the motifs they recognize (Desai et al., 2009; Huang et al., 2009; Camas et al., 2010; Leyn et al., 2011; Ravcheev et al., 2012). An important issue in such studies is to connect TFs to the cognate TF binding sites (TFBSs) identified by phylogenetic footprinting and other computational techniques (Conlan et al., 2005; Wels et al., 2006; Liu et al., 2008). This problem is either solved experimentally or addressed computationally, for instance for regulons controlled by local TF from specific protein families (Rigali et al., 2004; Francke et al., 2008; Sahota and Stormo, 2010; Ahn et al., 2012; Kazakov et al., 2013). Phylogenetic profiling of TF genes and motifs upstream of candidate regulon members is an alternative bioinformatics approach for assigning TFs to putative regulons (Rodionov and Gelfand, 2005). The comparative analysis of ligand-binding domains in TFs also helps identify ligand specificity determinants and propose models of functional diversification within large and functionally heterogeneous families of TFs (Kazanov et al., 2013).

Here we study the LacI family of bacterial transcription factors (LacI-TFs). The namesake of the family, the lactose repressor LacI of *E. coli*, has been the model object for the analysis of bacterial transcriptional regulation since the classical papers of Jacob and Monod (1959, 1961). The family was established by the analysis of similarity of protein sequences, and simultaneously the similarity of DNA motifs recognized by the family members was noted (Weickert and Adhya, 1992). At the same time, it was observed that its DNA-binding domains of LacI-family regulators are similar to the helix-turn-helix domains of other TFs (Nguyen and Saier, 1995), whereas the ligand-binding domain of LacI-TFs is homologous to the periplasmic proteins of ABC-transporters (Mauzy and Hermodson, 1992; Fukami-Kobayashi et al., 2003). Interestingly, this domain was also seen in combination with another DNA-binding domain, winged helix-turn-helix of the GntR family (Franco et al., 2006). While early observations on a limited number of sequences suggested that the history of the family involved a series of duplications at the early stage and a low level of duplications later on (Nguyen and Saier, 1995), more data, derived from bacterial genomes becoming available, demonstrated that the duplications were occurring throughout the history of the family (Fukami-Kobayashi et al., 2003). Given its large size and high level of structural similarity, yielding reliable multiple alignments, this family was widely used as a model for algorithms for identification of functionally important residues both uniformly conserved and specificity-determining (Mirny and Gelfand, 2002; Fukami-Kobayashi et al., 2003; Kalinina et al., 2004; Pei et al., 2006; Tungtur et al., 2007; Parente and Swint-Kruse, 2013). Some of these predictions were further tested in experiment (Meinhardt and Swint-Kruse, 2008; Camas et al., 2010; Tungtur et al., 2010, 2011).

The structural similarity of the DNA motifs recognized by the LacI-TFs was used to identify regulons in *Lactobacillus plantarum* (Francke et al., 2008) and *Dickeya dadantii* [*Erwinia chrysanthemi*] (Van Gijsegem et al., 2008). The structural aspects of interactions of the LacI-TFs with DNA and ligands and between subunits in dimers have been summarized in a recent review (Swint-Kruse and Matthews, 2009).

Here we report results of a large-scale, manual comparative genomics analysis of LacI-TFs aimed at identification of their binding sites, motifs, and regulons in bacterial genomes. By analyzing the genomic and metabolic context of the reconstructed regulons and by combining this analysis with information gathered from literature, we inferred the biological roles and molecular effectors for a large number of the studied LacI-TFs. As result, we made a number of observations on the distribution of orthologous LacI-TFs in genomes, the statistics of the binding sites' arrangement in regulatory regions, and the number and functional characteristics of the regulated genes. By combining the functional annotations with phylogenetic analysis, we proposed evolutionary models of functional diversification for a number of LacI-TF groups. The obtained reference dataset of 1281 regulons in 272 genomes was deposited in the RegPrecise database (Novichkov et al., 2013).

## Materials and methods

The genomes were downloaded from the MicrobesOnline database (Dehal et al., 2010). TFs from the LacI family were identified by similarity searches and domain predictions in the Pfam database (Finn et al., 2014). LacI-TFs consist of two characteristic domains, an N-terminal HTH DNA-binding domain (PF00356) and a C-terminal effector-binding domain, which is homologous to periplasmic binding proteins of sugar ABC transporters (PF00532, PF13377, or PF13407). Gene orthology was defined by the bidirectional best-hit criterion implemented in the GenomeExplorer software (Mironov et al., 2000) and validated by phylogenetic trees from the MicrobesOnline database (Dehal et al., 2010). Genes were considered as orthologs if they (i) formed a mono- or paraphyletic branch of the phylogenetic tree and (ii) demonstrated conserved chromosomal gene context. For genes in the reconstructed regulons, correspondence to both of these criteria was sufficient for these genes to be considered as orthologs. For the studied LacI-family regulators, an additional criterion of orthology was used. Thus, orthologous groups of LacI-TFs should (i) form a mono- or paraphyletic group on the phylogenetic tree; (ii) have a conserved gene context; (iii) have highly similar TFBS motifs; and (iv) have the same effector specificity (known or predicted based on the regulon content).

For the regulon reconstruction, we used a previously established comparative genomics approach (Rodionov, 2007) implemented in the RegPredict Web server (Novichkov et al., 2010). This approach includes prediction of putatively regulated genes, inference of TFBSs, construction of positional weight matrices (PWMs) for TFBS motifs, and a further search for additional regulon members on the basis of predicted TFBSs in gene promoter regions. Overall, three main strategies were used for the reconstruction of regulons: (1) construction of PWMs on the basis of known TFBSs, for regulons being previously analyzed in model organisms; (2) prediction of novel TFBS motifs in promoter regions of regulated genes, for regulons with only regulated genes but not TFBSs known; and (3) prediction of putatively co-regulated genes followed by the inference of putative TFBS motifs in their promoter regions and attribution of a candidate TF to a putative regulon. Presumably, regulated genes were predicted by the analysis of conserved gene neighborhoods around an analyzed LacI-TF gene. Data about known regulated genes and LacI-TFBS motifs were extracted from the literature and from the RegTransBase (Cipriano et al., 2013), RegulonDB (Salgado et al., 2013), DBTBS (Sierro et al., 2008), and CoryneRegNet (Pauling et al., 2012) databases.

Candidate motifs in upstream regions of regulated operons were identified by the Discover Profile tool in RegPredict (Novichkov et al., 2010). A search for palindromic DNA motifs of 14- to 24-bp length was carried out within putative promoter regions from −400 to +100 bp relative to the translational gene start. Motifs were further manually validated by phylogenetic footprinting, that is, analysis of conserved islands in multiple alignments of DNA fragments (Shelton et al., 1997). The constructed PWMs were further used to search for additional regulon members using the Run Profile tool in RegPredict. The lowest score observed in the training set of known and/or predicted TFBSs was used as the threshold for a site search in genomes. To eliminate false-positive TFBS predictions, the consistency check approach (Ravcheev et al., 2007; Rodionov, 2007) and/or functional relatedness of candidate target operons were used. In this approach, an operon can be considered as regulated when its upstream region contains putative TFBSs with a score higher than the threshold, and such sites can be found in a number of related genomes. Operons were defined as groups of genes satisfying the following criteria: same direction of transcription, intergenic distance up to 100 bp, absence of internal TF-binding sites, and conservation of the locus structure in a number of related genomes. All predicted TFs, motifs, and sites are available in the RegPrecise database (http://regprecise.lbl.gov/) (Novichkov et al., 2013), where they are publicly available within the TF family collections of regulons.

Functional gene annotations were extracted from the literature and uploaded from the SEED (Disz et al., 2010), UniProt (Magrane and Consortium, 2011), MicrobesOnline (Dehal et al., 2010), and KEGG (Kanehisa et al., 2012) databases. Known functional annotations for a particular gene were expanded to all orthologous genes. For prediction of gene functions, both the comparative genomics and context-based methods were used (reviewed in Osterman and Overbeek, 2003; Overbeek et al., 2005; Rodionov, 2007). Multiple alignments of protein and DNA sequences were built by MUSCLE (Edgar, 2004). Phylogenetic trees were constructed using a maximum-likelihood algorithm implemented in PhyML 3.0 (Guindon et al., 2010) and visualized via Dendroscope (Huson et al., 2007) and iTOL (Letunic and Bork, 2011). Sequence logos for DNA motifs were drawn with WebLogo (Crooks et al., 2004).

## Results

The comparative genomics workflow for regulon reconstruction implemented in the RegPredict Web server (Novichkov et al., 2010) and the RegPrecise database (Novichkov et al., 2013) includes three steps: (i) selection of a taxonomic group of related bacteria; (ii) selection of a subset of diverse genomes that represent a given group; and (iii) reconstruction of regulons in the selected genomes. For the analysis of LacI-TF regulons, we selected a set of 344 representative genomes from 39 taxonomic groups from 7 bacterial phyla (Table S1 in Supplementary Material). Among the analyzed lineages, there are 19 taxonomic groups of Proteobacteria (183 genomes), 9 groups of Firmicutes (72 genomes), and 7 groups of Actinobacteria (57 genomes). The Bacteroides, Chloroflexi, Deinococcus-Thermus, and Thermotogae phyla are each represented by a single taxonomic group and have 32 genomes in total.

### Repertoire of LacI-TF genes in bacterial genomes

To estimate the abundance of LacI-family TFs (LacI-TFs) in the studied genomes, we collected primary LacI-TF sets using a similarity search and the existing prokaryotic TF compilations. In total, 2572 proteins were found unevenly distributed in most (309/344; 90%) of the studied genomes, whereas 10% of the genomes do not encode putative LacI-TFs (Table S1 in Supplementary Material). The largest average numbers of LacI-TFs per genome were found in several lineages of the Actinobacteria phylum including Streptomycetaceae and Bifidobacteriaceae (from 32 and to 17 regulators), in two lineages of Proteobacteria–Rhizobiales and Enterobacteriales (15 regulators in each group), and in two lineages of Firmicutes–Bacillales and Enterococcaceae (12 regulators in each group). The remaining taxonomic groups possess less than 10 LacI-TFs per genome on average. Noteworthily, the Methylophilales, Neisseriales, Nitrosomonadales, Oceanospirillales, Magnetospirillum/Rhodosprillum, and Desulfovibrionales groups completely lack LacI-TFs in their genomes. The absence of LacI-TFs in these taxonomic groups of Proteobacteria can be related to (i) relatively small proportion of sugar catabolic genes in their genomes (as LacI-TFs mostly control sugar catabolism, see below); (ii) increasing usage of TFs from other families to compensate the contraction of the LacI-TF pool.

### Statistics of reconstructed regulons and regulogs

The entire set of identified LacI-TFs was broken into taxonomic group-specific orthologous groups that were subjected to further comparative genomics analysis using the RegPredict Web server (Novichkov et al., 2010). Normally, an orthologous group contained no more than one TF per genome. However, in some cases TFs formed by recent, mainly genome-specific, duplication were assigned to the same orthologous group (Table S1 in Supplementary Material). By analyzing orthologous groups of regulators in each taxonomic group, candidate motifs and binding sites were predicted for 1303 LacI-TFs (50% of all putative LacI-TFs) in 272 bacterial genomes (80% of studied genomes). The main outcome of this analysis is an annotated regulog, which is defined as a set of genome-specific regulons controlled by orthologous TFs. Overall, we inferred 1281 LacI-TF regulons that constitute 322 populated regulogs unevenly distributed across 39 studied taxonomic groups of genomes (Tables S1, S2 in Supplementary Material). The reconstructed regulons included 7465 candidate sites, 6076 operons, and 13,558 genes.

**Figure 1:**
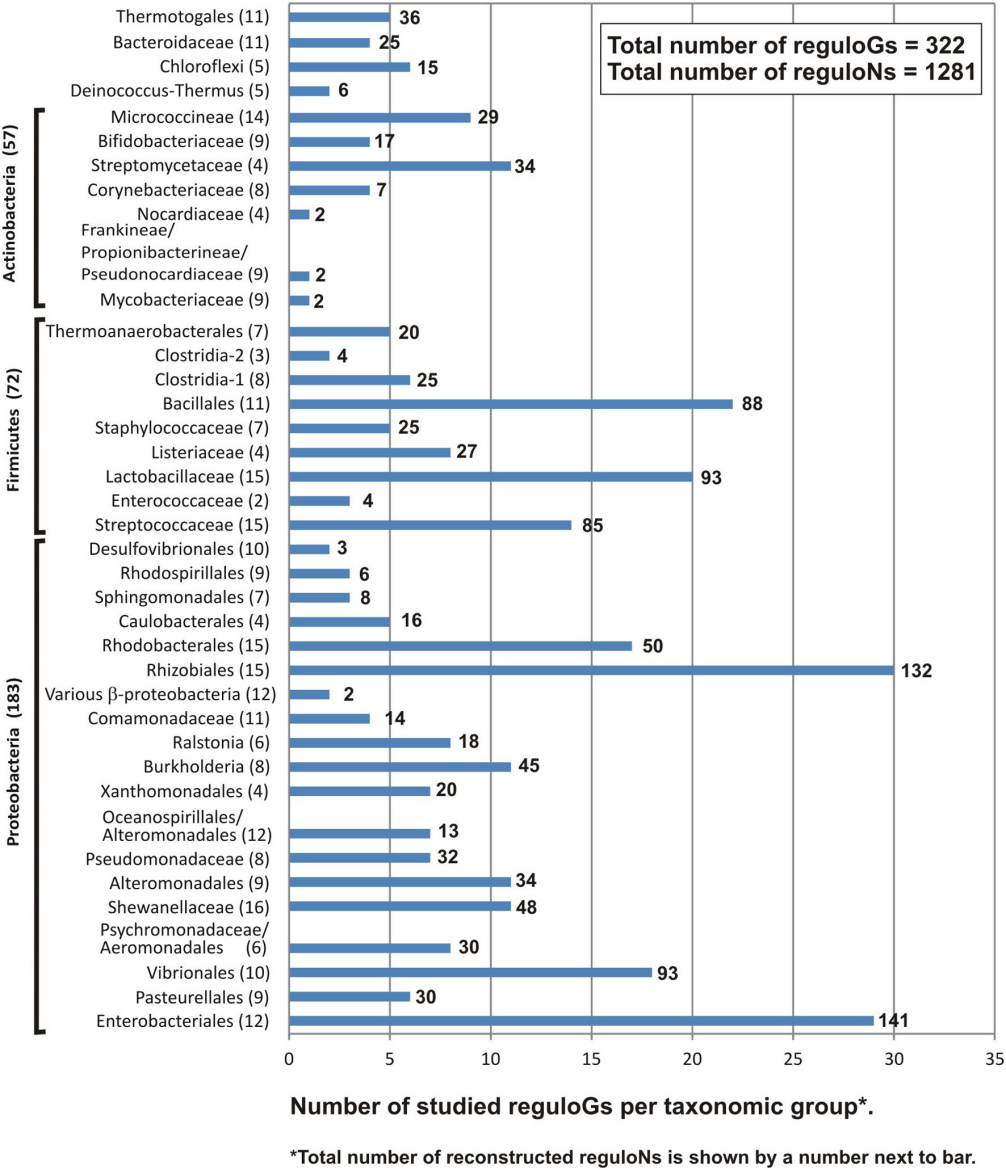
**Distribution of LacI-TF regulons and regulogs in the analyzed taxonomic groups of bacteria**.

The taxonomical distribution of the reconstructed LacI-TF regulons is highly uneven but generally follows the distribution of all LacI-family TFs (Figure 1): 57% of regulons were from Proteobacteria, about 30% from Firmicutes, 7% from Actinobacteria, about 1–2% from each of Thermotogales, Bacteroides, Chloroflexi, and Deinococcus/Thermus. Yet, compared to the genomic distribution of all putative LacI-TFs (Table S1 in Supplementary Material), the Actinobacteria phylum is underrepresented. Based on the phylogenetic analysis of LacI-TF proteins, regulators from the reconstructed regulogs were merged into larger orthologous groups that were consistent with the taxonomy, had regulated orthologous genes, and had similar binding motifs. TFs were assigned to an orthologous group if they formed a mono- or paraphyletic branch (see below) in the phylogenetic tree (Figure S1 in Supplementary Material). As in most gene families containing multiple paralogs resulting from frequent duplications, losses, and horizontal transfers, the resolution of orthology in some cases was difficult and required arbitrary decisions that are supported by the genomic context and/or functional attributes of the reconstructed regulons.

**Figure 2:**
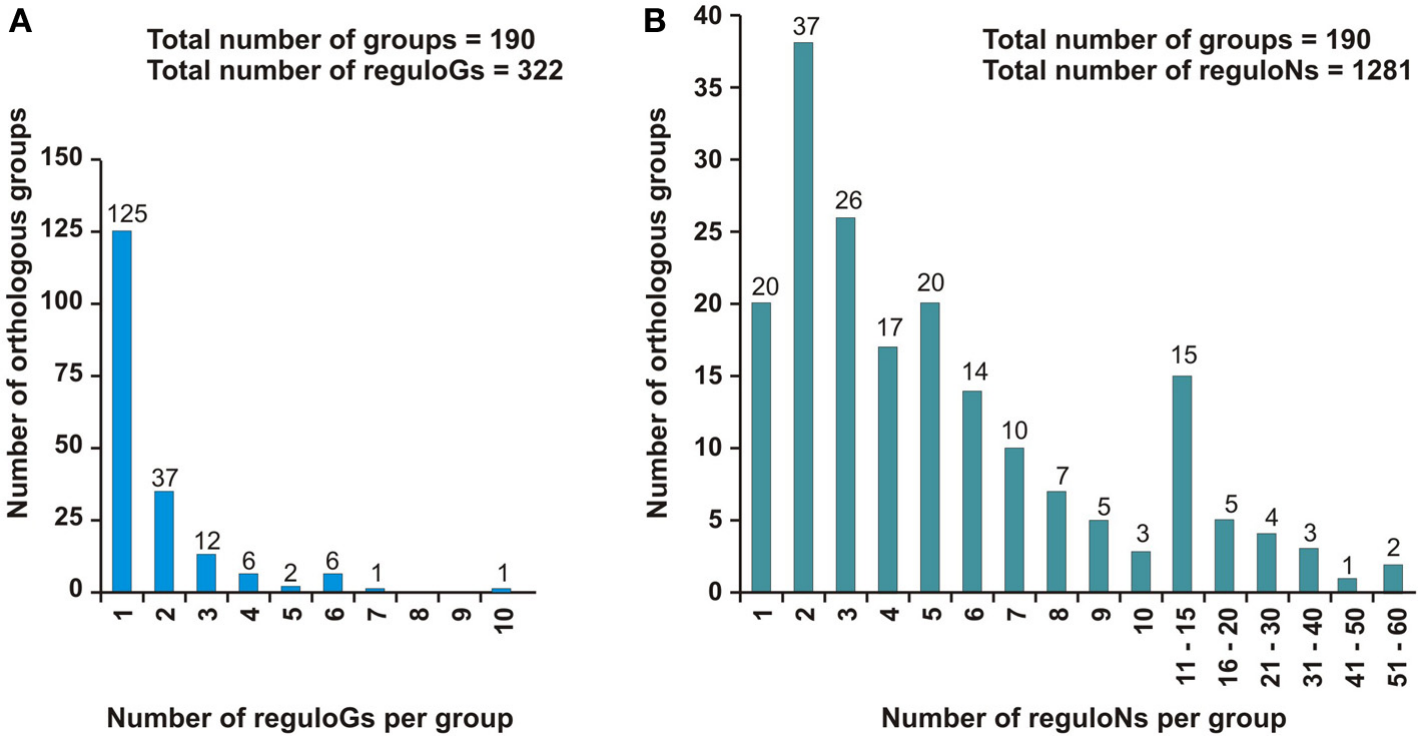
**Regulon and regulog content of the studied LacI-TF orthologous groups. (A)** Regulog content. **(B)** Regulon content.

As a result, the studied LacI-TFs were classified into 190 orthologous groups characterized by conserved DNA motifs and regulated pathways (Table S2 in Supplementary Material). Two-thirds of the obtained orthologous groups (125/190) contain TFs from a single regulog, which is defined as a set of orthologous regulons in a group of closely related genomes. Thirty-seven orthologous groups include two regulogs, whereas the remaining 28 groups were assigned to three or more regulogs (Figure 2A). The total number of regulons (and corresponding TFs) per orthologous group of LacI-TFs varies between 1 and 59, with the average being 6.7 (Figure 2B). The maximal number of groups was observed for the groups including two TFs (37 groups). Orthologous groups containing up to 5 and between 6 and 10 regulons constitute 60 and 20% of all groups, respectively. The most populated groups of LacI-TF orthologs were found for the global catabolite control regulator CcpA in Firmicutes (59 regulons, 6 regulogs), the ribose repressor RbsR in Proteobacteria (52 regulons, 10 regulogs), the maltose repressor MalR in Firmicutes (42 regulons, 6 regulogs), as well as sugar catabolism regulators FruR (40 regulons, 6 regulogs), GntR (37 regulons, 7 regulogs), and GalR (27 regulons, 5 regulogs) in γ-Proteobacteria.

### Global and local regulons

**Figure 3:**
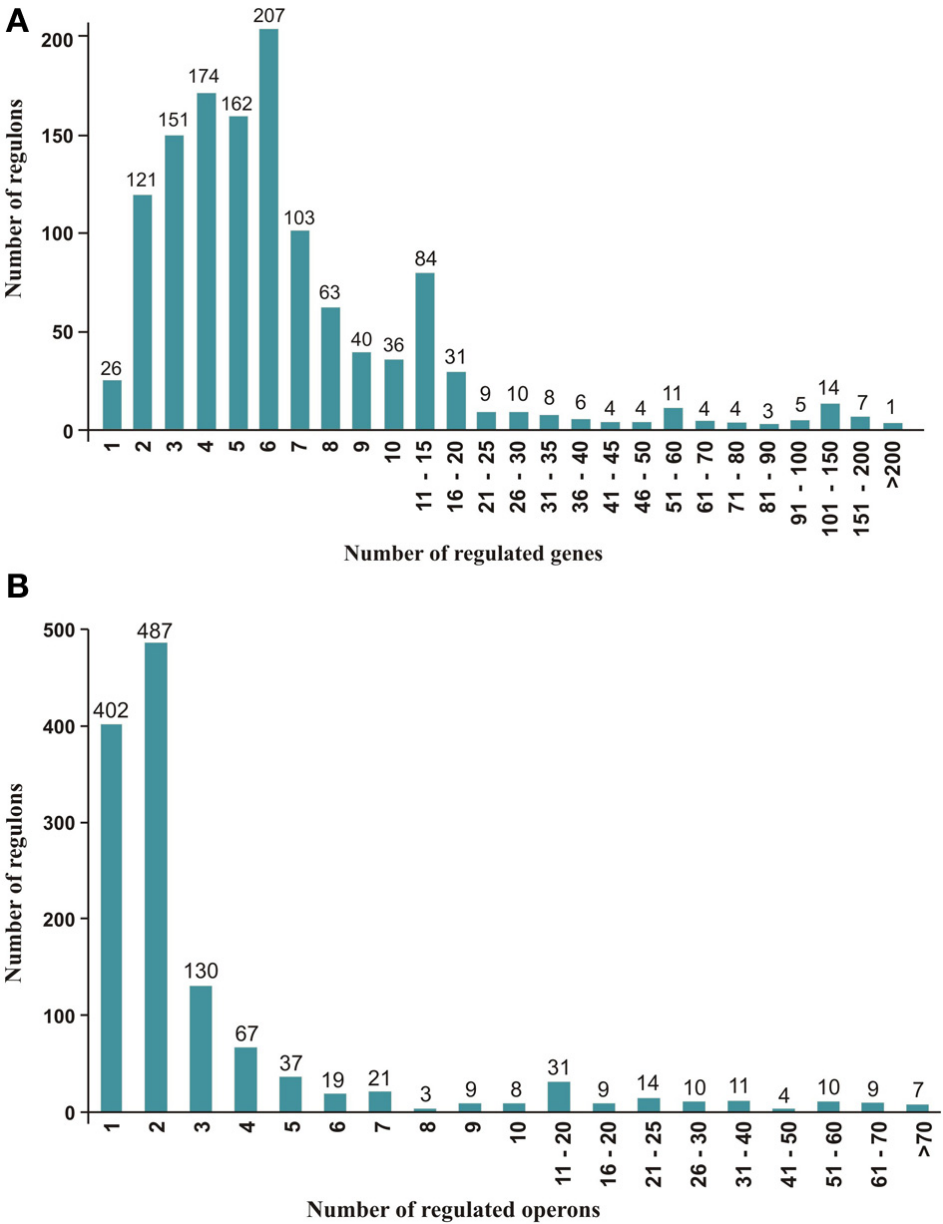
**Distribution of reconstructed LacI-TF regulons. (A)** Distribution by the number of regulated genes. **(B)** Distribution by the number of operons.

The reconstructed LacI-TF regulons demonstrate drastic differences in the numbers of predicted target genes and operons. The majority of regulons (1198/1288, 93%) include 20 or fewer genes (Figure 3A), and further, three-fourths of these regulons contain between 2 and 7 genes, whereas 26 regulons have only 1 target gene. With respect to the number of regulated operons (Figure 3B), the largest portion of the studied LacI-TFs regulates one (31%) or two (38%) operons. We divided all reconstructed LacI-TF regulons into two main categories depending on their size (number of regulated genes and operons) and functional diversity (number of regulated pathways). A total of 125 regulons (12 regulogs) were classified as global, since each of them (i) contained more than 15 target genes that were arranged in at least 7 operons and (ii) controlled multiple metabolic pathways. The remaining LacI-TF regulons (1163/1288, 90%) were classified as local, each having a smaller number of targets and controlling a single metabolic pathway, usually a particular carbohydrate catabolic pathway.

Almost one-half of the identified global regulons (59 regulons, 6 regulogs) are operated by orthologs of the *B. subtilis* catabolite control regulator CcpA in all analyzed taxonomic groups of Firmicutes. CcpA regulons were previously described in detail for bacteria from the Bacillaceae (Sonenshein, 2007; Fujita, 2009; Leyn et al., 2013), Staphylococcaceae (Seidl et al., 2009; Ravcheev et al., 2011), Lactobacillales (Mahr et al., 2000; Zheng et al., 2011, 2012; Zotta et al., 2012; Ravcheev et al., 2013b), and Clostridiaceae (Antunes et al., 2012) lineages. Another large group of global regulons (31 regulons, 3 regulogs) are operated by orthologs of the *E. coli* purine repressor PurR in three related taxonomic groups of γ-Proteobacteria—Enterobacteriales, Pasteurellales, and Vibrionales. PurR is a global transcriptional regulator of *E. coli*, controlling biosynthesis of purines, some steps of biosynthesis of pyrimidines, polyamine metabolism, and nitrogen assimilation (Ravcheev et al., 2002; Cho et al., 2011). The remaining three global regulogs—FruR in Enterobacterales, PckR in Rhizobiales, and GapR in Rhodobacterales (totaling 35 regulons)—that control the central and periphery carbohydrate catabolic pathways in diverse groups of Proteobacteria are described in more detail below.

Autoregulation was observed for 72% (943 TFs) of the studied LacI-TFs. The autoregulation of TFs was more typical for local regulons, as expected. Thus, less than one-half of the studied global regulators (55 TFs) demonstrated autoregulation, whereas more than three-fourths of the analyzed local regulators (888 TFs) were autoregulated.

### Binding sites and motifs

Binding motifs of the considered LacI-TFs are palindromes formed by highly conserved inverted repeats, which is consistent with previous studies (Francke et al., 2008; Camas et al., 2010; Milk et al., 2010). The distance between the repeats is usually constant for a given orthologous group of TFs, although in rare cases there is some flexibility. The overwhelming majority of LacI-TF binding motifs (1251/1303 TFs) are even palindromes (16, 18, 20, or 22 nt long), whereas non-canonical palindromes (17, 19, or 21 nt long) were found for only 4% of regulators (e.g., LacR, GalR, and EbgR). The characteristic feature of even palindromes is the presence of a consensus CG pair in the center of the palindrome (1173 TFs). Nonetheless, in some even palindromes the central pair can be either different (49 TFs) or degenerate (29 TFs).

**Figure 4:**
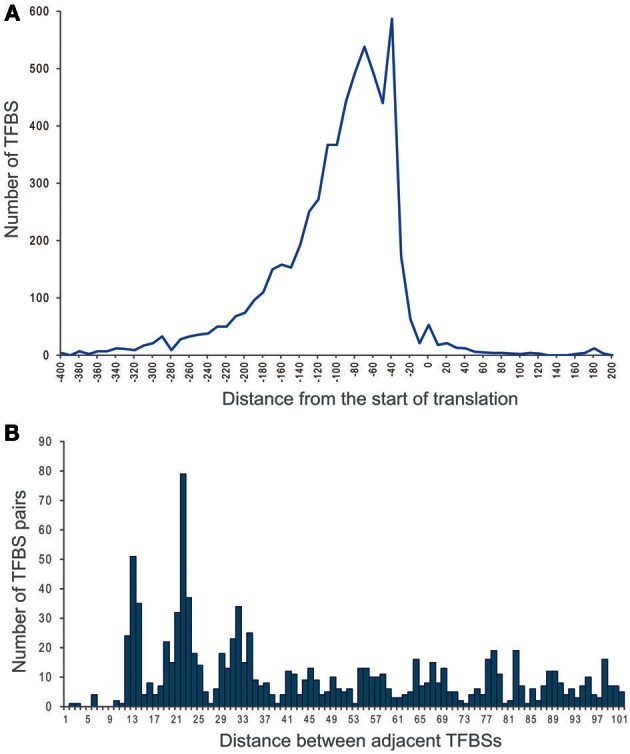
**Distribution of distances. (A)** Distances between LacI-TF binding sites and the translational start sites. **(B)** Distances between adjacent binding sites.

More than 75% of identified LacI-TFBSs are located within the area between 140 and 30 bp upstream of the start codon (Figure 4A). Less than 1% of sites are localized within coding regions, including experimentally demonstrated *E. coli* sites of LacI deep within the *lacZ* gene (Lewis, 2005) and PurR in the *purB* gene (He et al., 1992). Approximately 7% of sites are localized far upstream (>200 bp), and while some of them might in fact regulate divergently transcribed genes, experimental examples of such localization are known, e.g., the PurR site upstream of *prsA*, again in *E. coli* (He et al., 1993).

About 20% of regulated operons are preceded by more than one binding site. Thus, tandem sites were observed upstream of 1118 operons. The multiplicity of sites within regulatory regions of divergently transcribed genes (i.e., divergons) increases with the divergon length. Groups of three and four adjacent sites were found for 116 and 5 regulated operons, respectively.

The histogram of intersite distances for pairs of sites localized in the same intergenic region has pronounced peaks at 13, 22, and 32 bp (Figure 4B). The latter two lengths are multiples of the DNA helix step and hence are clearly indicative of cooperative binding of two TF dimers. Several LacI-TF regulogs, e.g., RafR from Enterobacteriales and ScrR from Burkholderiales, have only double sites at the distance of 21–22 bp, suggesting that the cooperative binding in this case is obligatory. Overlapping sites, situated at a distance of about 13 bp, were observed upstream of 110 operons. Such an arrangement may be functional, as has been demonstrated for GntR-binding sites upstream of *gntKU* of *E. coli* (Tsunedomi et al., 2003a) and it is conserved for more than one-half of the operons regulated by GntR and its orthologs in γ-Proteobacteria.

A trivial explanation—that these observations are an artifact—does not seem plausible for two reasons. Firstly, such multiple sites are conserved and even preferred in some orthologous groups. Secondly, the artifact hypothesis does not explain the preferred distance of 13 bp. In the available tertiary structures of LacI-TFs complexed with DNA (Schumacher et al., 1994; Barbier et al., 1997; Kalodimos et al., 2002), seven base-pairs nearest to the site center form contacts between bases and side residues. Hence, the site-overlap region strongly overlaps with the zone of specific TF-DNA contacts. While tetramer binding has been suggested, it is difficult to reconcile this with the structural data, as dimers bound at these distances cannot interact. It is possible that there exists a specific binding mode involving partial unwinding of the DNA strands.

### Regulated metabolic pathways and effectors

By assessing the functional content of the reconstructed regulons, we tentatively predicted possible biological functions and effectors for 190 orthologous groups of LacI-TFs. As result, metabolic pathways were predicted for 182 groups of LacI-TFs. These include 54 groups that were only assigned to the general category of sugar metabolism, and for them, a specific sugar catabolic pathway remained unknown (Table S2 in Supplementary Material). We compared the predicted regulon functions with previous results of experimental studies available for 24 selected LacI-TFs. The previously established functions of these regulators are in good agreement with the target-regulated pathways that were predicted in this work. Based on the metabolic pathway reconstruction and the knowledge of pathway metabolites, a range of possible molecular effectors was suggested for 108 groups of LacI-TFs (Table S2 in Supplementary Material). Of these, effectors were previously known for regulators from 21 LacI-TF groups.

As expected, the overwhelming majority of the studied LacI-TF orthologous groups control carbohydrate metabolism (176/182; 96% of groups with assigned pathways). At that, most of the orthologous groups containing local LacI-TFs are assigned to specific carbohydrate utilization pathways. In contrast, five orthologous groups of local regulators including AdeR, HpxR, and UriR control the nucleoside utilization pathways, whereas a local regulator NtdR controls the neotrehalosadiamine biosynthesis. Most global regulators from the LacI family also are involved in the control of carbohydrate metabolism (FruR, CcpA, PckR, GapR), whereas PurR regulates several key metabolic pathways including purine and pyrimidine biosynthesis.

In agreement with the observed tendency of LacI-TF to control sugar metabolism, we report that carbohydrates constitute the largest class of effectors for these regulators (103/107; 96% of orthologous groups with assigned effectors). The majority of carbohydrate effectors assigned to the LacI-TF groups are monosaccharides and their derivatives (26/45), including hexoses (e.g., glucose, galactose, mannose), pentoses (e.g., ribose, xylose), sugar phosphates (e.g., fructose-1-phosphate, allose-6-phosphate), sugar acids (e.g., gluconate, galacturonate), sugar alcohols (e.g., ribitol), and amino sugars (e.g., N-acetylglucosamine). The second-largest category of carbohydrate effectors contains various oligosaccharides (14/26), including common disaccharides cellobiose, maltose, sucrose, and trehalose or their phosphorylated derivatives (e.g., cellobiose-6-phosphate, sucrose-6-phosphate). Finally, non-carbohydrate effectors of LacI-TFs include nucleobases (e.g., guanine and hypoxanthine are co-repressors of PurR in *E. coli* Schumacher et al., 1994), nucleosides (e.g., cytidine and adenosine are inducers of CytR in *E. coli* Barbier et al., 1997), and proteins (e.g., phosphoprotein HPr-Ser46-P is a co-repressor of CcpA in *B. subtilis* Schumacher et al., 2011).

Functional analysis of reconstructed LacI-TF regulons revealed that many sugar utilization pathways are regulated by two or more non-orthologous regulators. These include catabolic pathways for at least 10 distinct types of carbohydrates that are controlled by more than 90 orthologous groups of LacI-TFs (Table 1; Table S1 and Figure S1 in Supplementary Material). The observed large numbers of non-orthologous regulators for the glucoside and galactoside catabolic pathways correlate with structural diversity of glucose- and galactose-containing oligosaccharides that can be utilized by bacteria in diverse natural habitats. On the other hand, the diversity of regulators for several other sugars including ribose and sucrose suggest a high frequency of convergent evolutionary events when the same ligand specificity has evolved independently in different branches of the LacI family.

**Table 1:** **Sugar utilization pathways controlled by non-orthologous LacI-TFs**.

**Sugar utilization pathway**	**LacI-TF orthologous groups**	
	**Number of groups**	**Regulator examples**
Glucose and glucosides	20	BglR, BglZ, CelR, AscG, KojR
Sucrose	11	CscR, ScrR, SuxR
Galactose and galactosides	10	BgaR, GalR, GanR, MsmR, EbgR, LacI, LacR
Ribose	9	RbsR
Maltose and maltodextrins	8	MalR, MdxR, MalI
Inositol	7	IolR
Gluconate and idonate	7	GntR, IdnR
Glucuronate and galacturonate	6	ExuR, KdgR, UxaR, UxuR
Mannose and mannosides	5	ManR
Fructose and fructooligosaccharides	5	FruR, BfrR
Trehalose	4	TreR, ThuR

**Figure 5:**
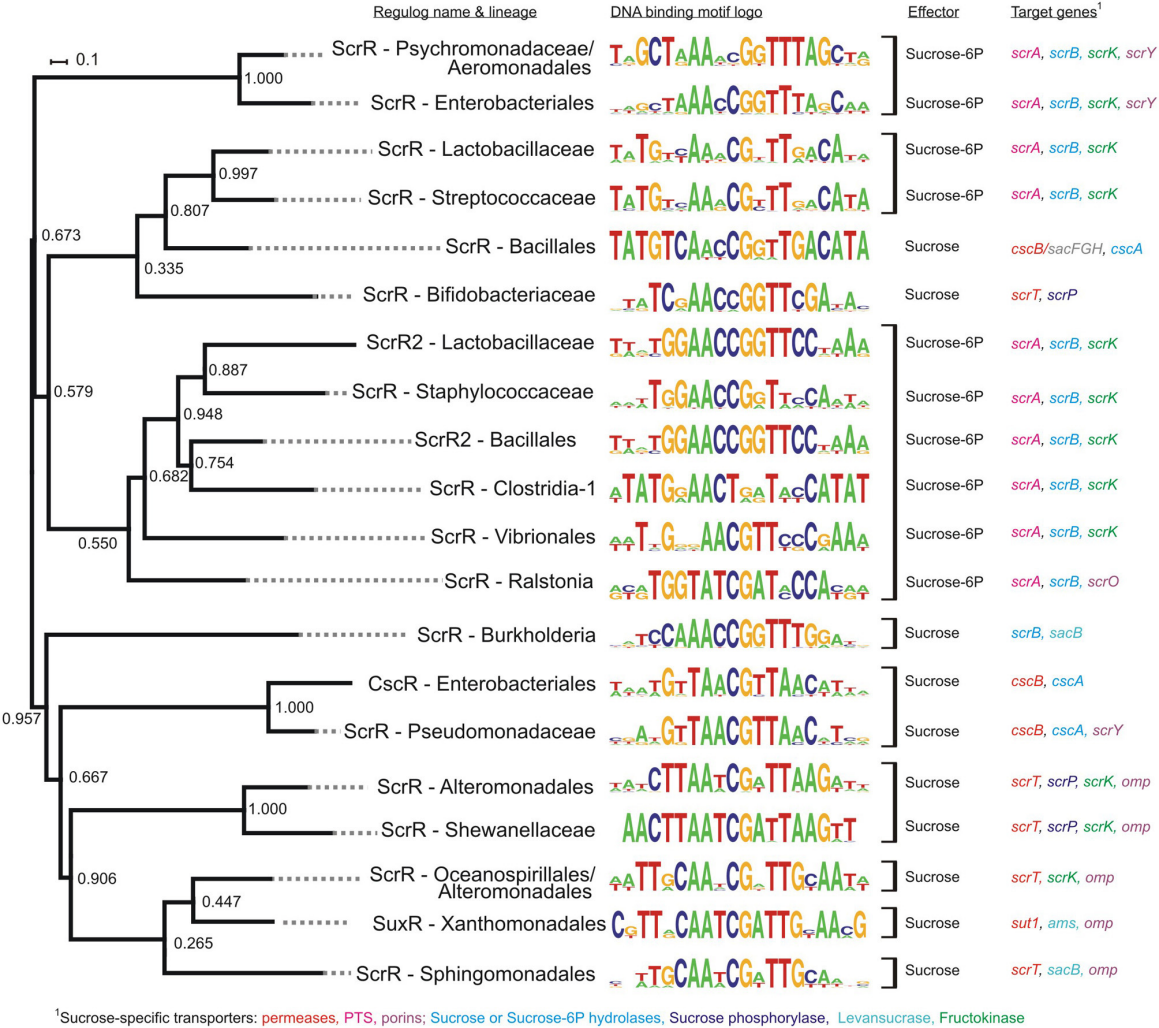
**Phylogenetic tree, binding site logos, effectors, and regulon content for regulators of sucrose utilization**. Orthologous groups of TFs are shown by square brackets.

For example, the sucrose utilization pathway is regulated by LacI-TFs from at least 11 orthologous groups from the phyla of Proteobacteria (11 lineages) and Firmicutes (5 lineages), as well as a single lineage of Actinobacteria (Figure 5). Analysis of the respective sucrose regulons revealed multiple distinct combinations of sucrose uptake transporters including permeases (*scrT*, *cscB*, *sut1*), phosphotransferase systems (PTSs) (*scrA*) and porins (*scrY*, *scrO*, *omp*), and sucrose catabolic enzymes including phosphorylases (*scrP*), hydrolases (*scrB*), and fructokinases (*scrK*). The effectors sucrose and sucrose-6-phosphate were assigned to the respective groups of ScrR regulators based on the type of regulated sucrose-specific transporters, i.e., a permease or a PTS, respectively. Interestingly, some families such as Bacillales and Enterobacteriales contain non-orthologous ScrR regulators with different effectors. As expected, DNA motifs of TFs controlling the sucrose catabolic pathway are well conserved within orthologous groups but are clearly different between non-orthologous regulators (Figure 5).

### Global regulons for central sugar metabolism

The LacI family contains a number of global regulators for central carbohydrate metabolic pathways, in particular the previously known regulators CcpA in Firmicutes and FruR in Enterobacteria. Here, we report a comparative genomics reconstruction of orthologous FruR regulons in γ-Proteobacteria, while the reconstructions of CcpA regulons in different lineages of Firmicutes has been reported previously (Ravcheev et al., 2011, 2013b; Antunes et al., 2012; Leyn et al., 2013) and is available in the RegPrecise database. Further, we report identification of three novel non-orthologous regulons (named PckR, GapR, and GluR) that control central carbohydrate metabolism in three lineages of α-Proteobacteria, namely, Rhizobiales, Rhodobacterales, and Caulobacterales. Below we provide functional analysis of regulons for each of these four LacI-family regulators in Proteobacteria.

**Figure 6:**
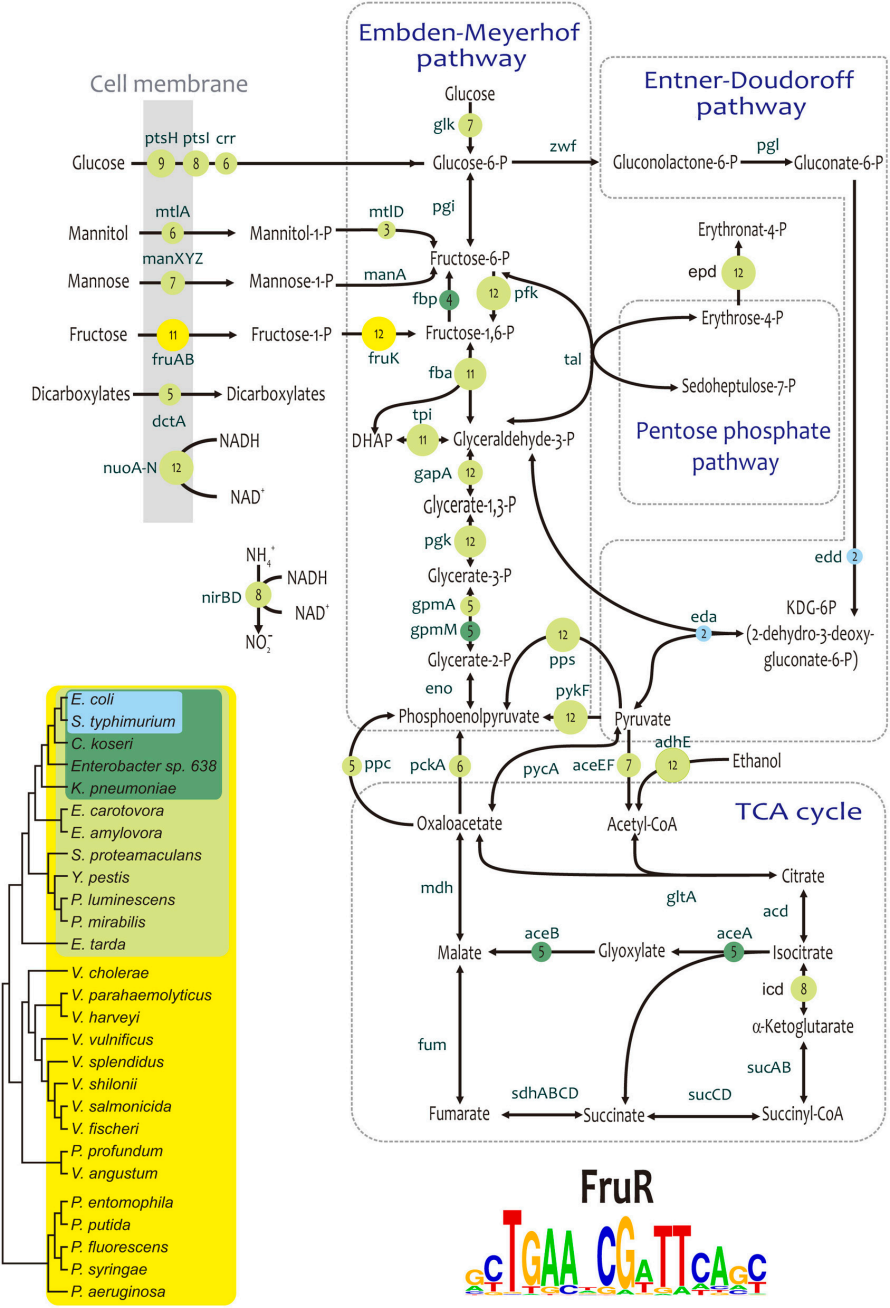
**Content of reconstructed FruR regulons in Gammaproteobacteria**. Names of regulated genes and operons are shown at arrows. Numbers in circles show the numbers of genomes with correspondent regulation.

The fructose repressor FruR (also known as the catabolism repressor and activator Cra) is a global regulator of central metabolism in *E. coli* (Saier and Ramseier, 1996). FruR/Cra coordinates the carbon flow by repressing glycolytic genes involved in the Embden–Meyerhof, Entner–Doudoroff, and pentose–phosphate pathways and by activating gluconeogenesis genes. The comparative genomics reconstruction of orthologous FruR regulons in γ-Proteobacteria revealed that the regulon size correlates with the taxonomy of studied groups and with the phylogeny of the FruR proteins (Figure 6). In Vibrionales and Pseudomonadales, the fructose utilization operon *fruBKA* and the *fruR* gene are the only members of the reconstructed FruR regulons; therefore, FruR operates as a local regulator in these lineages. In the Enterobacteriales, the FruR regulon is expanded to cover genes of the central glycolytic pathways, a part of the TCA cycle, and several fermentation and respiration pathways. Further regulon expansion to the genes of glyoxylate bypass is observed in the *Escherichia*, *Salmonella*, *Citrobacter*, *Enterobacter*, and *Klebsiella* species. Finally, in the closely related *Escherichia* and *Salmonella* species, the FruR/Cra regulon is expanded to include the Entner–Doudoroff pathway genes. Similar trends in the evolution of global regulons in closely-related bacterial species were previously demonstrated for the PhoP regulon in enterobacteria (Perez and Groisman, 2009). On the other hand, in Pasteurellales, the regulon is degrading: FruR is absent in the *Haemophilus* spp. and *Actinobacillus pleuropneumoniae*, whereas in *Pasteurella multocida*, *Mannheimia succiniciproducens, A. succinogenes*, and *A. aphrophilus* the *fruR* gene is seemingly intact, but no candidate FruR-binding sites could be detected.

**Figure 7:**
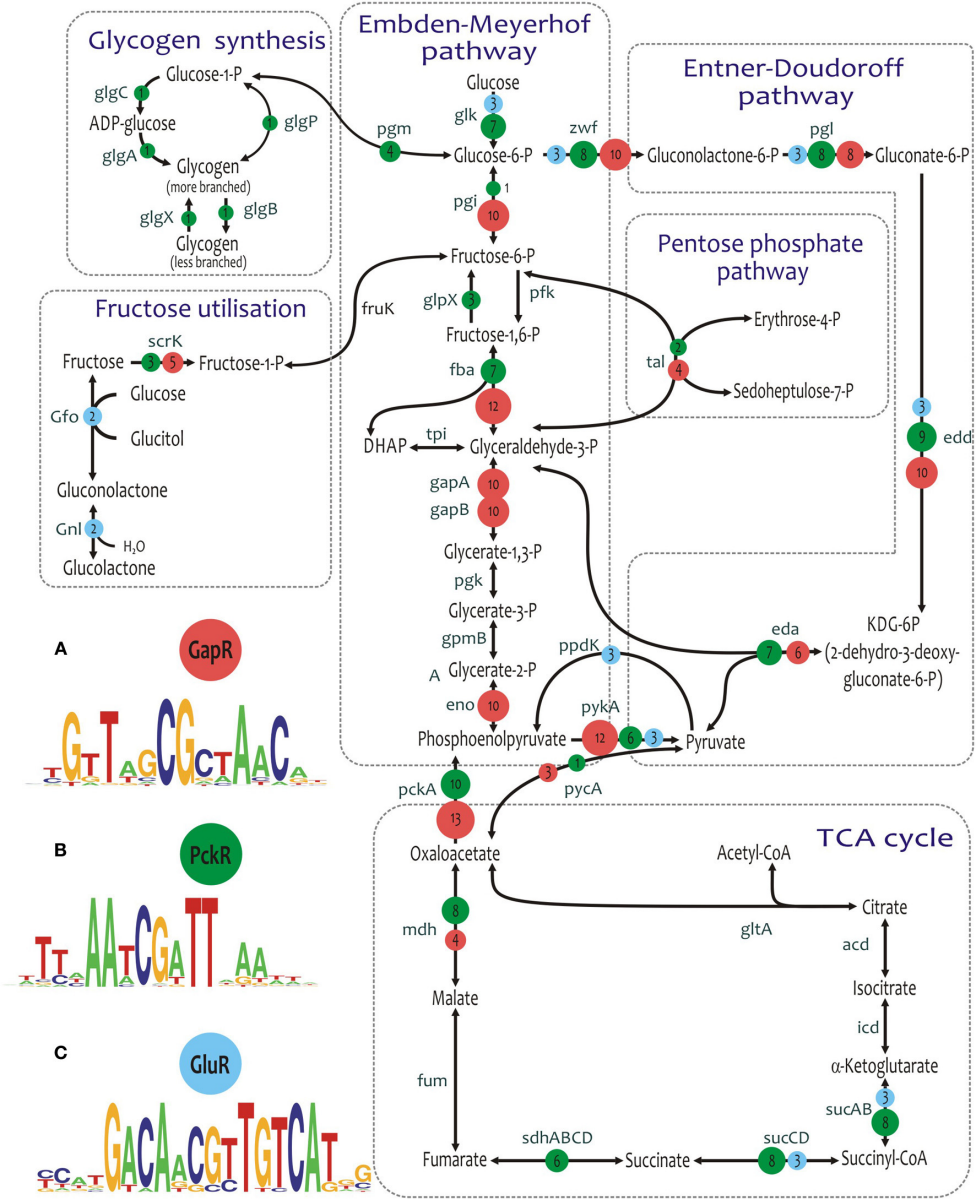
**Predicted global regulons for central carbohydrate metabolism in Alphaproteobacteria. (A)** GapR regulon in Rhodobacteraceae. **(B)** PckR regulon in Rhizobiales. **(C)** GluR regulon in *Caulobacter* spp. Names of regulated genes and operons are shown at arrows. Numbers in circles show the numbers of genomes with correspondent regulation.

The hypothetical TF PckR (SMc02975 in *Sinorhizobium meliloti*) was previously annotated as a putative regulator of the phosphoenolpyruvate carboxykinase *pckA* (EMBL accession number AF004316.1); however, it had not yet been studied experimentally. Orthologs of PckR were found in 10 out of 15 analyzed genomes from the Rhizobiales order including species from the Rhizobiaceae, Brucellaceae, Phyllobacteriaceae, and Xanthobacteraceae families. By using the comparative genomics approach, we identified the putative PckR binding motif and reconstructed the PckR regulons in each of these genomes (Figure 7). Furthermore, by analyzing the binding-site position within promoter regions, we predicted a negative or positive mode of PckR regulation. As result, PckR was predicted to function as a dual transcriptional regulator that represses glycolytic genes from the Embden–Meyerhof and Entner–Doudoroff pathways (*glk*, *fba*, *pykA*, *zwf-pgl-edd*, *eda*) and activates genes from the gluconeogenesis and TCA cycle (*pckA*, *mdh-sucCDAB*, *sdhABCD*).

In the Xanthobacteraceae family, the reconstructed PckR regulon includes a minimal number of genes (*pckA*, *pckR*, *edd*, and *hpr*), whereas it is significantly expanded in the other three families of Rhizobiales and contains from 14 to 28 genes per genome. The most conserved members of the reconstructed PckR regulons include the *pckA* and *edd* genes (in 10 and 9 genomes, respectively), the *zwf-pgl* and *mdh-sucCDAB* operons (in 8 genomes), and the *fba*, *glk*, and *eda* genes (in 7 genomes). In most of the analyzed genomes, the *pckR* genes are not clustered with their target genes, which is a common feature of many global regulators in bacteria. However, in the genome of *Xanthobacter autotrophicus*, *pckR* is divergently transcribed with the 6-phosphogluconate dehydrogenase gene *gnd*, which is preceded by a putative PckR-binding site.

Orthologs of PckR and their cognate DNA motifs were found in Rhizobiales but not in other lineages, suggesting that the PckR regulon was introduced relatively recently in the evolution of α-Proteobacteria. PckR from Rhizobiales can be considered as a partial functional replacement of the Enterobacterial Cra/FruR (see above) and the *Shewanella* HexR regulators (Leyn et al., 2011) that both play a pleiotropic role modulating the direction of carbon flow through different carbohydrate metabolic pathways. The molecular effector of PckR is unknown. A plausible hypothesis is that PckR dissociates from its DNA sites in response to an intermediate of the central glycolytic pathway in rhizobia.

A novel global transcriptional regulon for carbohydrate metabolism genes named GapR (RSP_1663 in *Rhodobacter capsulatus*) was identified in all 13 studied genomes from the Rhodobacteraceae family (Figure 7). GapR was predicted to recognize a 20-bp palindromic DNA consensus, which is distinct from the PckR-binding consensus. The reconstructed GapR regulons contain from 7 to 18 genes per genome organized in 5–13 operons. GapR regulates glycolytic genes involved in the Embden–Meyerhof and Entner–Doudoroff pathways (*zwf-pgl-pgi*, *edd-eda*, *fba*, *gapA*, *gapB*, *eno*, *pykA)*, gluconeogenesis (*pckA*, *pycA*), fructose utilization (*scrK*), pentose phosphate pathway (*tal*), and the TCA cycle (*mdh*). A similar DNA motif was identified upstream of the *gapR* genes in five genomes, suggesting their autoregulation. Similarly to PckR in Rhizobiales, the *gapR* genes do not cluster on the chromosome with their target genes in most genomes. However, *Roseobacter* possess two copies of *gapR*. One of these paralogs is divergently transcribed with the *gapB* gene, which is preceded by a putative GapR-binding site. The molecular mechanism and effector for GapR regulators remain to be elucidated.

Although PckR and GapR regulate overlapping sets of genes from the central carbohydrate metabolism in two distinct lineages of α-Proteobacteria, these regulators are not orthologous to each other (Figure S1 in Supplementary Material) and recognize different DNA motifs (Figure 7). Another non-orthologous regulator from the LacI family named GluR (CC2053 in *Caulobacter crescentus*) was predicted to control the central carbohydrate metabolism in the Caulobacteraceae family (Figure 7). GluR recognizes a conserved 20-bp palindromic consensus, different from the PckR- and GapR-binding motifs above. The reconstructed GluR regulon in the *Caulobacter* spp. is composed of the glycolytic genes *zwf-pgl-edd-glk*, *pykA*, and *gnl-gfo*, as well as the gluconeogenic gene *ppdK* encoding pyruvate-phosphate dikinase and the *sucABCD* operon involved in the TCA cycle. The predicted regulatory gene *gluR* is located immediately downstream of the target operon *zwf-pgl-edd-glk* and is preceded by a conserved GluR-binding site in all three analyzed *Caulobacter* genomes.

Orthologs of GluR were not found in other α-Proteobacteria, although bacteria from the Caulobacterales lineage have three paralogs (BglR1-3) that are predicted to control the cognate operons involved in the β-glucoside utilization (Figure S1 in Supplementary Material). The molecular effector of GluR is not known. Based on its close similarity to the predicted BglR repressors that possibly respond to β-glucosides and/or glucose, we propose that GluR dissociates from its DNA sites in response to glucose. In confirmation of our hypothesis, it has been demonstrated that glucose induces expression of the Entner–Doudoroff pathway genes and that the *edd* and *glk* genes are essential for the glucose utilization in *C. crescentus* (Hottes et al., 2004).

## Discussion

We used the comparative genomics reconstruction of regulons for analysis of the LacI family of bacterial TFs. This choice was based on the following features. First, the LacI family is large, varied, and broadly distributed in bacteria. Second, proteins from this family have a rigid domain structure and a highly conserved structure of TFBS motifs. This study resulted in a detailed reconstruction of 1281 LacI-TF regulons in 272 bacterial genomes. Most (~90%) of the analyzed TF-LacI regulons are local, i.e., they control a small number of genes and operons that are involved in only one metabolic pathway. However, some LacI regulons are global, controlling tens to hundreds of genes involved in multiple metabolic pathways. In addition to the reconstruction of previously known global regulons, such as FruR/Cra, PurR, and CcpA, we identified three novel regulators for the central carbohydrate metabolism in α-Proteobacteria, PckR, GluR, and GapR, and reconstructed their corresponding regulons. For two of these global regulons, FruR and GluR, we reconstructed their possible evolutionary histories. Both these TFs likely originated from local regulators during a process of gradual regulon expansion.

A large-scale phylogenetic analysis of LacI-TFs reveals numerous examples of various evolutionary processes for regulators and their regulons including divergent evolution (diversification of TF functions and binding specificities after duplication), convergent evolution (appearance of the same function in distantly related branches of a phylogenetic tree), and formation of paraphyletic groups (origin of novel functions and specificities, non-characteristic for a given branch of TFs). Below we discuss these evolutionary processes in more detail and provide examples of functional diversification within the LacI-TF family.

**Figure 8:**
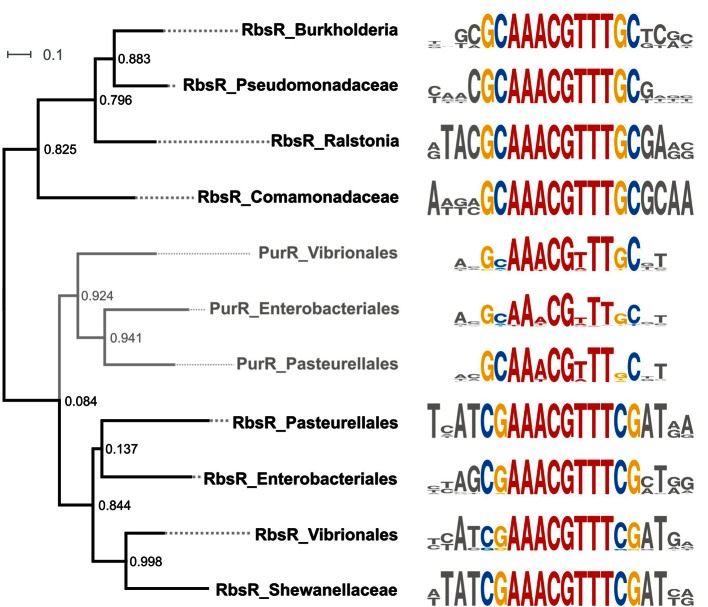
**Binding site motifs for PurR and RbsR from Beta- and Gammaproteobacteria**. Positions conserved for all motifs are shown in red; branch specific positions are shown in blue (consensus C) and yellow (consensus G); non-conserved positions are shown in gray.

The LacI-TF phylogenetic tree demonstrates that some orthologous groups of TFs form branches consistent with the taxonomy, with TFs regulating orthologous genes, and with recognizing similar motifs, but those branches included an internal clade with demonstrated differences in the motif structure, effector specificity, or regulon content, i.e., these TFs formed so-called paraphyletic groups. The most interesting example of paraphyly is the branch of ribose repressors RbsR in β- and γ-Proteobacteria that have the purine repressor PurR as an excluded clade. Phylogenetic analysis revealed the presence of PurR orthologs in only three bacterial orders, Enterobacteriales, Pasteurellales, and Vibrionales. The closest PurR paralogs in these groups are ribose repressors (RbsRs) (Figure S1 in Supplementary Material). Most probably, PurR was originated by duplication of RbsR in the common ancestor of Enterobacteriales, Pasteurellales, and Vibrionales. The Pseudomonadales and β-Proteobacteria with a single RbsR repressor seemingly feature the ancestral state. The RbsR orthologs from the above three orders retain the ligand specificity but have a slightly modified DNA binding motif, compared to RbsR of Pseudomonadales and β-Proteobacteria (Figure 8). On the contrary, PurR retained the motif but changed the ligand and the default state, as it binds DNA in the presence of its ligand, whereas RbsR, like the majority of the LacI-TFs, binds DNA in the absence of the ligand.

This example illustrates a possible way of formation of paraphyletic branches by duplication of an ancestral gene with subsequent conversion of one copy of the copies, resulting in the origin of a novel function. In the case of RbsR and PurR, duplication and conversion are rather ancient, and we observe only the result of such evolution but cannot observe the intermediate states of the evolutionary process. On the contrary, for the α-glucoside utilization regulon AglR and the trehalose regulon ThuR, such an intermediate state is clearly observable. AglR regulators from Rhizobiales and Rhodobacteriales form a paraphyletic branch with ThuR regulators from Rhizobiales as an excluded clade (Figure S1 in Supplementary Material). Both AglR and ThuR are local regulators, each controlling expression of the regulator genes and one other operon, divergently transcribed with the latter. The regulated operons contain homologous genes for kinases and ABC transporters, but non-homologous genes for hydrolases. The TFBS motif for ThuR (natcnAAAnCGnTTTngatt) is different from the one for AglR (nnntcAAAGCGCTTTgannn). Thus, during diversification, ThuR changed both the ligand and motif specificity. For the paraphyletic group CelR in γ-Proteobacteria, the AscG regulator in Enterobacteriales is an excluded clade. In the case of the CelR and AscG regulators, their TFBS motifs and sets of regulated genes are drastically changed after duplication, but the effector specificity (cellobiose-6P) and the target metabolic pathway (cellobiose utilization) are retained.

The phylogenetic tree for the analyzed LacI-TFs (Figure S1 in Supplementary Material) is patchy, complicating the reconstruction of the evolutionary history. However, in some cases we can observe two or more adjacent branches, each corresponding to one orthologous group. A natural explanation is that these groups appeared as a result of initial duplication of a TF gene followed by diversification of copies. The LacI-TF tree contains multiple examples of monophyletic taxon-specific branches that consist of proteins from a single bacterial lineage. One such branch includes the *Bacteroides* UxaR, UxuR, and KdgR regulators that control the catabolic pathways for galacturonate, glucuronate, and 2-keto-3-deoxygluconate, respectively. Two other monophyletic branches include (i) kojibiose regulator KojR and the unknown sugar regulator Caur_3448 from *Chloroflexus* and (ii) ribose regulator RbsR and uridine regulator UriR from *Corynebacteria* spp. Previously, a similar situation was observed for the ROK family of sugar-specific regulators in the deeply branched Thermotogales lineage (Kazanov et al., 2013).

Other examples of divergent evolution of regulator specificity are often demonstrated by adjacent branches in the phylogenetic tree. Thus, idonate repressor IdnR in Enterobacteriales and gluconate repressor GntR in multiple lineages of γ-Proteobacteria are the closest paralogs (Figure S1 in Supplementary Material). In the IdnR regulons, the *idnK* and *idnT* genes are the closest homologs of the GntR-regulated genes *gntK* and *gntU*, respectively. Thus, duplication affected not only a TF gene, but also some of the regulated genes. The ability of GntR to recognize IdnR-binding sites upstream of the *idnK* and *idnDOTR* operons in *E. coli* (Tsunedomi et al., 2003b) also confirms a recent duplication of these regulators. Structural similarity of sugar effectors for GntR and IdnR also points to a recent duplication and further specialization of IdnR in Enterobacteriales.

Another scenario of TF diversification is duplication of an ancestral TF gene followed by acquisition of novel regulated genes and, accordingly, new effector specificity. An example is provided by a branch containing fructose (FruR) and sucrose (ScrR) orthologous groups in γ-Proteobacteria. Sucrose is a fructose-containing disaccharide. Because the FruR- and ScrR-regulated genes are functionally and structurally different, the regulator was most probably duplicated alone, and then one copy acquired new regulated genes. The acquisition of a novel regulatory function was coupled with the changes in the cognate TFBS motif. Here, divergent TFs have structurally similar effectors, fructose-1,6-biphosphate for FruR and sucrose-6-phosphate for ScrR.

Based on the analysis of paraphyletic branches and adjacent monophyletic branches, three main types of the origin of TF with novel functions can be described. The first type is duplication of both the TF gene and a regulated gene or operon followed by diversification, as in the case of ThuR in Rhizobiales. During diversification, the TF and regulated genes change their specificities, some regulated genes may be lost, and novel genes may be included in the regulon. The second type is duplication of a regulator followed by acquisition of novel regulated genes, as for PurR in γ-Proteobacteria or for AscG in Enterobacteriales. The third type is rare, with only one example—the SCO5692 regulon in Streptomycetaceae. In this case, novel specificities for TFs originated without duplication, probably resulting from the loss of regulated genes.

In the process of acquisition of a new function, three characteristics of a TF regulon can be changed: (i) a set of regulated genes, (ii) effector specificity, and (iii) a TFBS motif structure. In most cases of TFs with a novel function, we observed the change of at least two of these characteristics. Change of all three characteristics is rarer and is usually observed in TFs from deeply branched lineages of bacteria such as Thermotogales. Most probably, in these cases change of all three characteristics is a result of a long evolutionary history. Change of only one of these characteristics is observed for only recently duplicated TFs, for which the diversification process is not yet complete.

In summary, the obtained extensive dataset for the LacI-TF family provides numerous examples of various evolutionary processes for regulators and their regulons. These data are publicly available in the RegPrecise database within the LacI family collection, which will enable further detailed analysis of signature residues in both DNA- and ligand-binding domains of regulators and establishment of the correlations between these residues and specificities toward the DNA motifs and molecular effectors they recognize.

## Author contributions

Dmitry A. Rodionov, Pavel S. Novichkov, and Mikhail S. Gelfand conceived and designed the research project. Dmitry A. Ravcheev, Dmitry A. Rodionov, and Mikhail S. Gelfand wrote the manuscript. Dmitry A. Ravcheev, Dmitry A. Rodionov, Matvei S. Khoroshkin, Olga N. Laikova, Olga V. Tsoy, Natalia V. Sernova, and Svetlana A. Petrova performed comparative genomics analysis to reconstruct TF regulons. Dmitry A. Rodionov provided the quality control of annotated regulons in the RegPrecise database. Matvei S. Khoroshkin analyzed statistical properties of TF regulons. All authors read and approved the final manuscript.

### Conflict of interest statement

The authors declare that the research was conducted in the absence of any commercial or financial relationships that could be construed as a potential conflict of interest.
